# Study on the Macroscopic Properties and Microstructure of High Fly Ash Content Alkali-Activated Fly Ash Slag Concrete Cured at Room Temperature

**DOI:** 10.3390/ma18030547

**Published:** 2025-01-25

**Authors:** Zhu Yuan, Yanmin Jia, Xuanben Xie, Junming Xu

**Affiliations:** 1 School of Civil Engineering and Transportation, Northeast Forestry University, Harbin 150040, China; zhu_yuan163@163.com (Z.Y.); xjm_2021@126.com (J.X.); 2 Qingdao Highway Development Center, Qingdao 266075, China; xuanbenxie01@126.com

**Keywords:** alkali-activated fly ash slag concrete, room temperature curing, high fly ash content, impermeability performance, water absorption curve, microstructure

## Abstract

Fly ash and granulated blast furnace slag are both bulk industrial solid wastes. Using these two raw materials to completely replace cement and prepare alkali-activated fly ash slag concrete (AAFSC) at room temperature can not only efficiently utilize industrial solid waste and reduce the carbon footprint, but also reduce the economic cost and technical difficulty of construction, which is of great significance for promoting the sustainable development of the concrete industry. In this article, the content of fly ash accounted for 80% of the total precursor (fly ash + slag), and a mixed solution of sodium silicate and sodium hydroxide was used as alkali activator to prepare AAFSC by curing at room temperature. The effects of alkali equivalent and activator modulus on compressive strength, impermeability, water absorption, and microstructure were systematically studied and compared with ordinary Portland cement concrete. The conclusions drawn were as follows. The 7-day compressive strength of AAFSC was lower than that of cement concrete, while its 28-day compressive strength was 104.86% to 131.94% of that of cement concrete. AAFSC exhibited excellent impermeability protection performance. The water absorption rate of AAFSC was lower, with A8M1 having a water absorption rate of 2.13%, which was only 60.86% of cement concrete. Through microscopic analysis, it was found that the alkali-activated fly ash slag cementitious matrix had good bonding with the aggregate, and there existed fly ash particles with different degrees of reaction. The Ca/Si value of AAFSC was smaller than that of cement concrete.

## 1. Introduction

As bulk industrial solid waste, fly ash production in China reached 899 million tons in 2023, accounting for about 55% of the global fly ash production. The total production of granulated blast furnace slag was over 60 million tons. These typical bulk industrial solid wastes are currently underutilized due to technological constraints, lack of standards, application market concepts, and transportation costs. They still need to occupy farmland, forest land, and grassland for stacking, which damages the ecological environment and awaits further industrial reuse or environmentally friendly treatment.

Due to the potential volcanic ash activity of fly ash and slag, they can serve as precursors for alkali-activated materials. By using alkali-activated technology, AAFSC can be prepared and applied in the construction of transportation infrastructure such as highways and bridges. It is expected to consume a large amount of fly ash and slag, while also reducing the production of traditional cement, which has significant environmental benefits.

By reason of the high activation energy of fly ash, pure fly ash-based alkali-activated cementitious materials require high-temperature curing to accelerate the reaction rate [1,2]. However, high-temperature curing requires excessive energy consumption, such as electricity. Compared with ordinary cement concrete, high-temperature curing increases the difficulty of construction technology and construction costs, which are difficult for developers, designers, and builders of engineering projects to accept. It will reduce the applicability of AAFSC in civil engineering construction. Therefore, achieving room-temperature curing can reduce the resistance to using AAFSC as a new type of concrete material in engineering.

Previous studies have shown that alkali-activated fly ash slag cementitious materials and concrete prepared by adding slag to pure fly ash could achieve satisfactory workability, setting time, and compressive strength under ambient-temperature curing [3,4]. One of the reasons was that, compared with the spherical shape of fly ash, slag particles have an angular shape and react more quickly. Adding slag could significantly affect the room-temperature setting performance of concrete [5]. The combination of slag and fly ash was superior because slag contributed to early strength, while fly ash contributed to later strength [6].

At present, some research results have been obtained on alkali-activated fly ash slag cementitious materials. Studies have investigated the effects of mixing time and mixing process [7,8], additives [9,10], curing conditions [11], fly ash microspheres [12], fly ash sources, surface area [13], and other factors on the mechanical, rheological, and microscopic properties of alkali-activated fly ash slag cementitious materials.

Research on AAFSC has found that AAFSC with different fly ash contents was suitable for different optimal curing schemes (water curing and air curing) [14]. To achieve good mechanical properties, lower porosity, and microcracking rates, appropriate fly ash/slag ratios, water, and admixture contents were required [15]. With the increase of slag content and activator, the durability performance of alkali-activated concrete such as freeze–thaw resistance increased [16,17], but the setting time decreased accordingly [18]. The sulfate resistance test showed that alkali-activated materials also have strong resistance to sodium sulfate corrosion [19]. By utilizing non-destructive testing techniques [20,21,22], rapid detection of damage to composite materials such as alkali-activated concrete can be achieved, providing more possible experimental methods for evaluating the development of internal defects. Using fly ash and slag instead of cement to prepare alkali-activated recycled aggregate concrete, denser alkali-activated cementitious materials could improve the shortcomings of recycled aggregate and the interface transition zone, thereby making alkali-activated recycled aggregate concrete have good mechanical performance [23,24]. The study on the alkali silica reaction in AAFSC showed that increasing the fly ash content in the alkali-activated fly ash slag system would reduce the expansion of concrete prisms containing medium-to-high-activity aggregates [25]. Reducing the porosity was conducive to the durability of concrete. By studying the influence of plasticizers on the refinement of pore structure of alkali-activated concrete and paste mixture, it was found that admixtures were most likely to improve the gel polymerization product layer around the slag particles, making the matrix more compact [26].

Through the above review, it is found that although there have been some research results on AAFSC, the proportion of fly ash was relatively small, which was not conducive to the bulk utilization of fly ash. Moreover, there is still a lack of systematic research on the mechanical properties, durability, and microstructure of AAFSC.

The AAFSC prepared in this study increased the fly ash content to 80% of the precursor dosage and was cured under room temperature and sealed conditions, using 20% slag to promote reaction rate and improve early strength of the concrete. We systematically studied the effects of activator modulus and alkali equivalent on the properties of AAFSC, including compressive strength, air permeability, water absorption performance, and microstructure. The ordinary cement concrete prepared in the research had the same amount of cementitious material and aggregate as the AAFSC. A systematic comparative analysis was conducted on the performance of AAFSC and cement concrete, providing a research basis for the engineering application and promotion of the AAFSC.

## 2. Raw Materials and Mix Proportions

### 2.1. Raw Materials

In the precursor of AAFSC, the fly ash (Harbin Shuangda Fly Ash Products Factory, Harbin, China) used was first-grade fly ash with a density of 2.30 g/cm^3^; the chemical composition is shown in Table 1. The grade of the slag (Hebei Jinghang Mineral Products Co., Ltd., Shijiazhuang, China) used was S95, with a density of 2.90 g/cm^3^ and a specific surface area of 418 m^2^/kg. The chemical composition of slag is shown in Table 2.

The alkaline activator was a mixed solution of sodium silicate (Tongxiang Hengli Chemical Co., Ltd., Jiaxing, China) and sodium hydroxide (Inner Mongolia Junzheng Energy Chemical Group Co., Ltd., Wuhai, China). In liquid sodium silicate, the content of silicon dioxide was 32.35%, the content of sodium oxide was 13.73%, the modulus was 2.43, the density was 1.53 g/mL, and the water content was 53.92%. Sodium hydroxide was a sheet-like solid with a purity of not less than 96%. The fine aggregate was natural river sand, medium sand, and the coarse aggregate was 5–20 mm continuously graded crushed stone. For detailed information on aggregates, please refer to the author’s previous research [28]. The water-reducing agent used was a naphthalene-based water-reducing agent (Shandong Wanshan Chemical Co., Ltd., Weifang, China).

### 2.2. Mix Proportions

In the mix proportions of AAFSC, the content of fly ash was 80% of the total precursor. When the fly ash content was high, a lower activator modulus and a higher activator dosage were necessary for the good development of strength [29,30]. Three different alkali equivalents were set, 4%, 6%, and 8%, and three different activator moduli were set, 1.0, 1.2, and 1.4. The detailed mix proportions are shown in Table 3.

The cement concrete used for comparative analysis had the same total amount of cementitious materials, sand ratio, and aggregate content as AAFSC. Fly ash, slag, and silica fume were used to replace a portion of cement in binder to achieve better performance. The mix proportion of cement concrete are shown in Table 4.

## 3. Specimen Preparation and Curing

**Figure 1:**
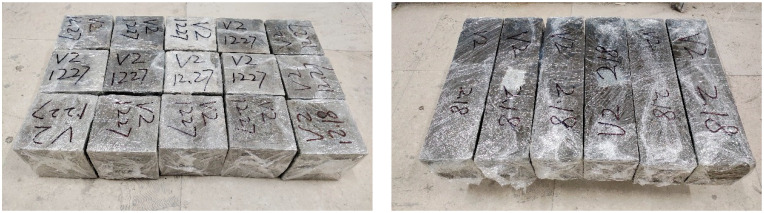
AAFSC specimens cured at room temperature.

After mixing the concrete raw materials evenly, they were placed in the mold and compacted with a vibration table (the vibration time was approximately 1 min). At room temperature (20 °C), specimens were kept in the mold for 24 h. After demolding, the AAFSC specimens were tightly wrapped with cling film to prevent moisture evaporation, and then were cured at room temperature (20 °C) to the specified age. This study prepared two specimens of different sizes: cube specimens of 100 mm in side length and prism specimens with dimensions of 100 mm × 100 mm × 400 mm, as shown in Figure 1. The cement concrete was placed in a standard curing room for water curing until the specified age.

## 4. Test Procedures

### 4.1. Compressive Strength Test

According to the standard GB/T 50081-2019 [31], The experimental instrument was a YAW-2000A computer-controlled electro-hydraulic servo universal tester (made by Jinan Time Shijin Testing Machine Co., Ltd., Jinan, China). The loading rate was 0.8 MPa/s. A total of 60 cubic specimens 100 mm in side length were used to test the 7-day and 28-day compressive strength of concrete. Each group tested three samples, and the average value was taken to reduce the test error.

### 4.2. Air Permeability Test

**Figure 2:**
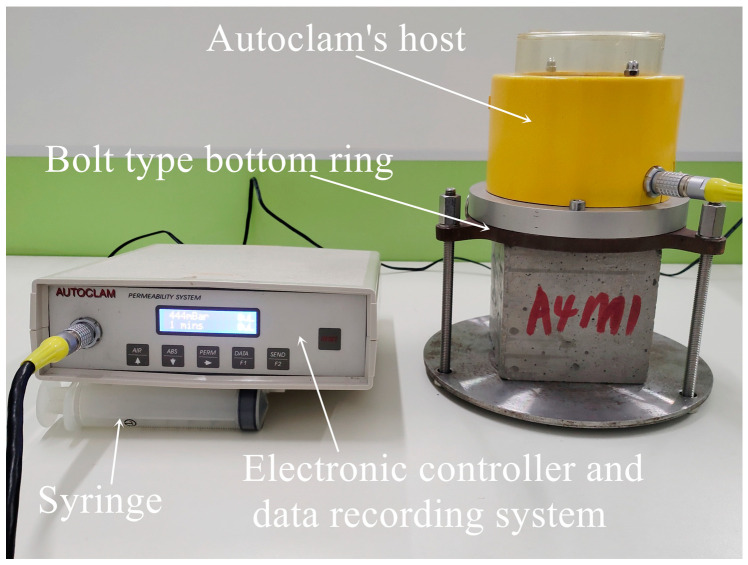
The Autoclam permeability system.

The instrument used to test the air permeability of concrete specimens was the Autoclam permeability system (Autoclam, amphora NDT, Belfast, Northern Ireland, UK), as shown in Figure 2. During the test, a standard silicone ring with an inner diameter of 50 mm was used to isolate the test area on the surface of the concrete specimen, and the pressure in the test area was increased to 500 mbar with a syringe. As the air dissipated on the surface of the test area, the pressure decreased. If the air dissipated too quickly, the pressure would drop to zero within 15 min.

The formula for calculating the air permeability coefficient Ka (m^2^) was as follows:

Ka (m^2^) = (API)^0.8754^ × 8.395 × 10^−16^ (1)

where API was the air permeability index, and its unit was Ln (Pressure)/min.

A total of 30 cubic specimens 100 mm in side length were used to test the air permeability of the AAFSC and cement concrete. Three samples were tested in each group, and the average value was taken as the result.

### 4.3. Water Absorption Test

**Figure 3:**
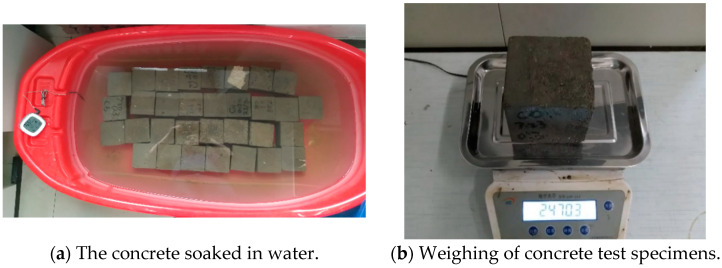
Water absorption test of concrete.

A total of 30 cubic specimens 100 mm in side length were used to test the water absorption properties of AAFSC and cement concrete. As shown in Figure 3a, the full immersion water absorption test was carried out in the water tank. According to the standard ASTM C642-2013 [32], the weight of concrete after water absorption was continuously recorded, as shown in Figure 3b, and the full water absorption curves of AAFSC and cement concrete were obtained. Three samples were tested in each group, and the average value was taken as the experimental results.

### 4.4. Microstructure Analysis Experiment

**Figure 4:**
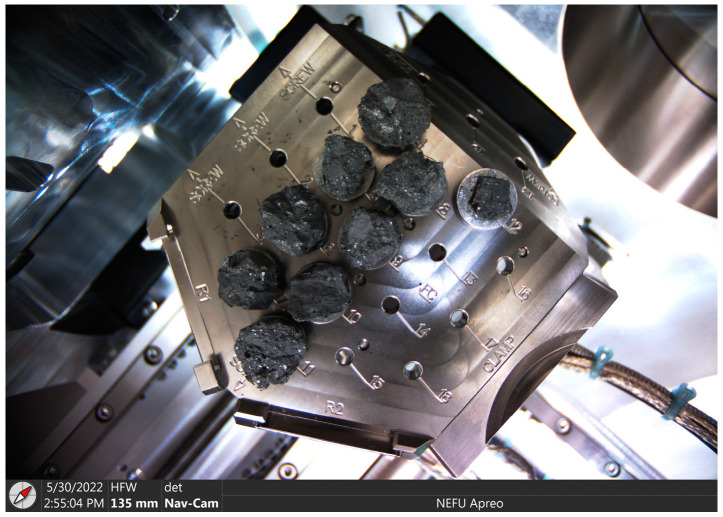
Concrete samples placed on the SEM scanning workbench.

The SEM test procedure was conducted according to the standard ASTM C1723-2010 [33]. Cubic cement concrete and AAFSC specimens 100 mm in side length were used for SEM observations. After curing for 28 days, fresh cross-section samples were taken from inside the specimens for microscopic observation. The SEM and EDX experiments were carried out using an Apreo SEM (made by ThermoFisher Scientific, Waltham, MA, USA). Figure 4 shows the concrete samples after surface gold spraying treatment placed on the SEM scanning workbench.

## 5. Results and Discussion

### 5.1. Compressive Strength

**Figure 5:**
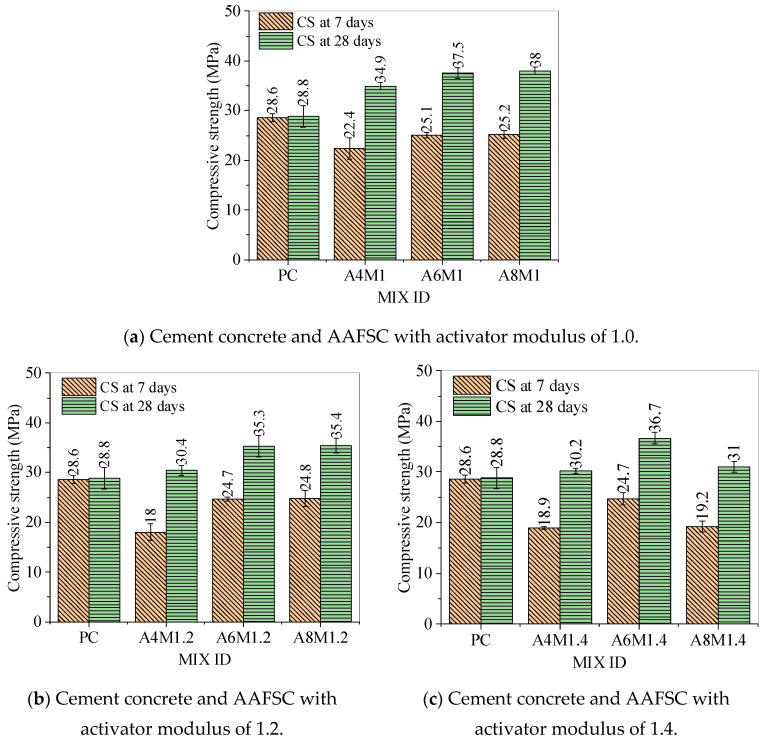
Compressive strength of concrete.

Table 5 and Figure 5 show the compressive strength of AAFSC and cement concrete. When the modulus of alkali activator was 1.0 and 1.2, the 7-day and 28-day compressive strengths of AAFSC increased with the increase of alkali equivalent. The 28-day compressive strengths of A8M1 and A8M1.2 were 38.0 MPa and 35.4 MPa, respectively. When the modulus of alkali activator was 1.4, the compressive strength first increased and then decreased with the increasing alkali equivalent.

The compressive strengths of cement concrete at 7 and 28 days were 28.6 MPa and 28.8 MPa, respectively. No significant increase in compressive strength of cement concrete was observed between 7 and 28 days, which might be due to the addition of supplementary cementitious materials requiring a longer curing time than 28 days to achieve suitable strength. The compressive strength range of AAFSC at 7 days was from 18 MPa to 25.2 MPa, all lower than that of the cement concrete. Due to the high content of fly ash, under normal-temperature curing, the reaction rate of AAFSC was relatively slow in the first 7 days. However, with the extension of curing time, the reaction of alkali-activated cementitious materials continued. By the 28th day, the compressive strength range of the nine groups of AAFSC was from 30.2 MPa to 38 MPa, and the strength values were all higher than that of cement concrete.

### 5.2. Air Permeability

The measurement of air permeability depends on the porosity, pore geometry, pore curvature, and most importantly, pore connectivity of the material. It is an important method for characterizing the microstructure and transport characteristics of materials, and is also one of the important indicators for measuring the service life and durability of concrete [34,35].

**Figure 6:**
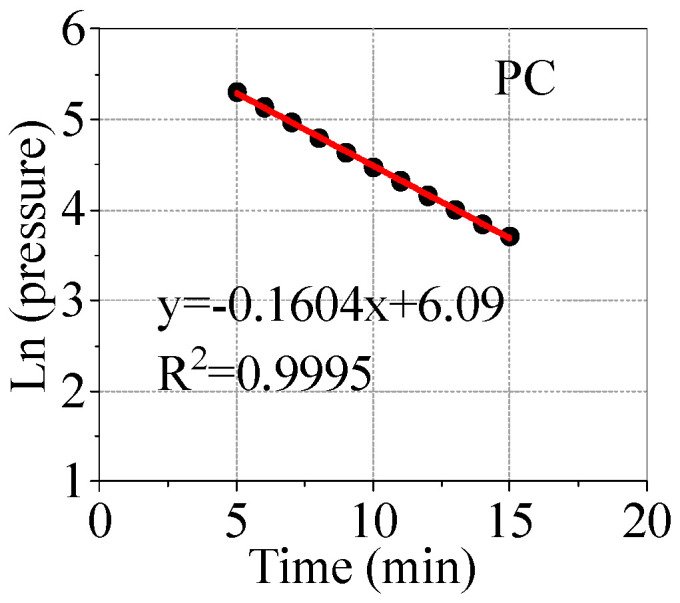
The relationship between the natural logarithm of the air pressure value of cement concrete and time.

**Figure 7:**
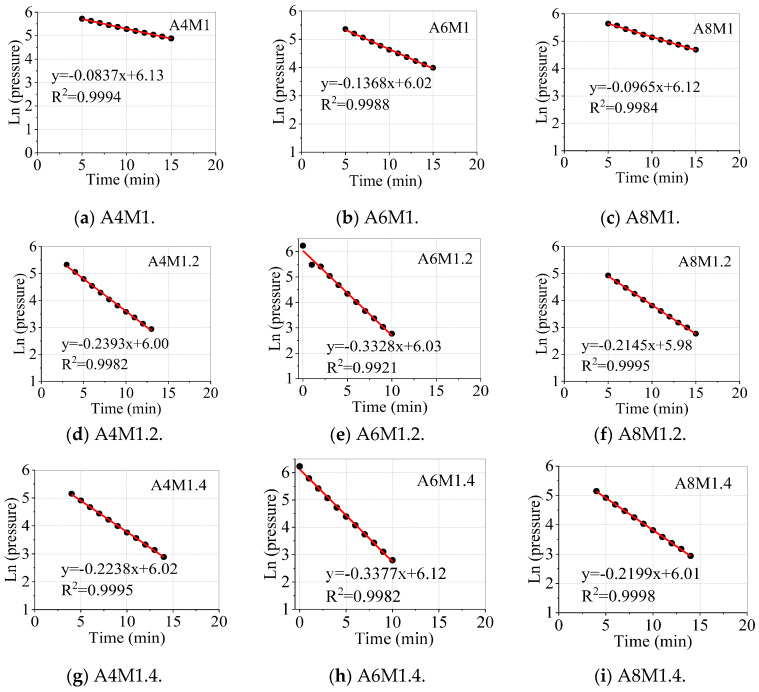
The relationship between the natural logarithm of the air pressure value of AAFSC and time.

During the impermeability test of cement concrete and AAFSC, the natural logarithm of the air pressure value in the standard silicone ring isolation area and its relationship with time are shown in Figure 6 and Figure 7, respectively. In the figure, the absolute value of the slope of the fitting curve corresponding to the test data points represents the Air Permeability Index (API). The larger the API value, the faster the pressure drop in the standard silicone ring isolation area, and the poorer the impermeability of the concrete.

Table 6 shows the air permeability coefficient of concrete and the quality level of concrete impermeability protection evaluated based on it. The air permeability coefficient of AAFSC first increased and then decreased with the increasing alkali equivalent, and increased with the increase of activator modulus. Compared with cement concrete, it can be seen that AAFSC with an activator modulus of 1.0 had better impermeability than cement concrete. Among them, A4M1 had the smallest air permeability coefficient, which was 0.957 × 10^−16^, indicating the best impermeability performance.

Overall, the impermeability performance protection quality level of AAFSC was “good” or “very good”, indicating that AAFSC had good impermeability performance.

### 5.3. Water Absorption Analysis

The water absorption rate could be used to characterize the volume and connectivity of pores in concrete, and is a key parameter affecting the durability of concrete, such as frost resistance [36,37,38,39,40,41].

**Figure 8:**
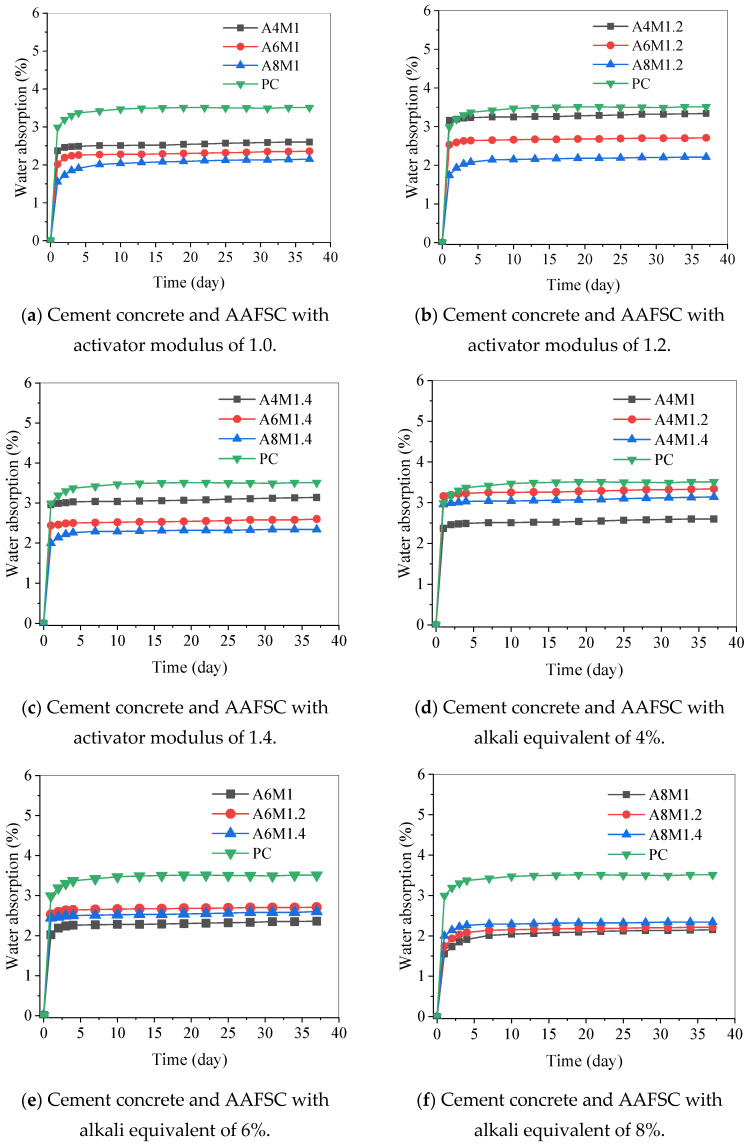
Water absorption curves of cement concrete and AAFSC.

According to the trend of water absorption of the concrete specimens, the test data were collected until the 37th day. Figure 8 shows the full curves of water absorption of AAFSC and cement concrete. All of the water absorption curves of AAFSC with different alkali equivalents and activator moduli were below the cement concrete, indicating that its water absorption rate was lower than that of cement concrete. This means that in AAFSC, the carrier for transporting corrosive substances such as chloride ions and sulfate ions will be reduced, delaying the deterioration process of the internal structure of AAFSC and enhancing its durability [30,35].

When the activator modulus was determined, the water absorption rate decreased with the increase of alkali equivalent. When the alkali equivalents were 4% and 6%, respectively, as the activator modulus increased, the water absorption first increased and then slightly decreased. When the alkali equivalent was 8%, the water absorption rate increased with the increasing activator modulus, and the water absorption rate of AAFSC with a modulus of 1.0 was even lower. Compared with the results of compressive strength, it can be seen that as the alkali equivalent increased, the trend of water absorption was generally opposite to that of compressive strength.

The above analysis shows that the water absorption rate of concrete followed an exponential distribution over time. Based on the nonlinear relationship between the water absorption rate and time, this paper established an exponential water absorption model that could describe the long-term water absorption process of concrete:

(2) $$A_{w}=\varphi \left(1-e^{-\omega t}\right)$$

where $A_{w}$ is the water absorption rate (%), φ and ω are the material parameters, and t is the time (day).

The water absorption model parameters for different types of concrete were obtained, as shown in Table 7. This model could accurately reflect the nonlinear relationship between the water absorption rate and time of AAFSC and cement concrete.

### 5.4. Microscopic Analysis

**Figure 9:**
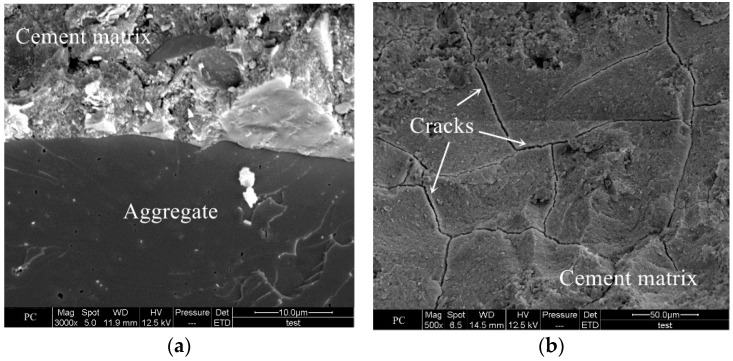
SEM images of cement concrete. (**a**) Bonding between aggregate and cement matrix. (**b**) Cracks on the surface of cement matrix.

Figure 9 shows the SEM images of the cement concrete specimen. In Figure 9a, the coarse aggregate was tightly bonded to the cement matrix, and the cement matrix structure was dense. However, it can be observed from Figure 9b that there were cracks on the surface of the cement matrix when magnified 500 times. The reasons for the formation of these cracks, on the one hand, may be caused by shrinkage during the hydration process of cement, and on the other hand, the sample observed by SEM was taken from a cube specimen 100 mm in side length, which might cause cracking of the cement matrix during the sampling process.

**Figure 10:**
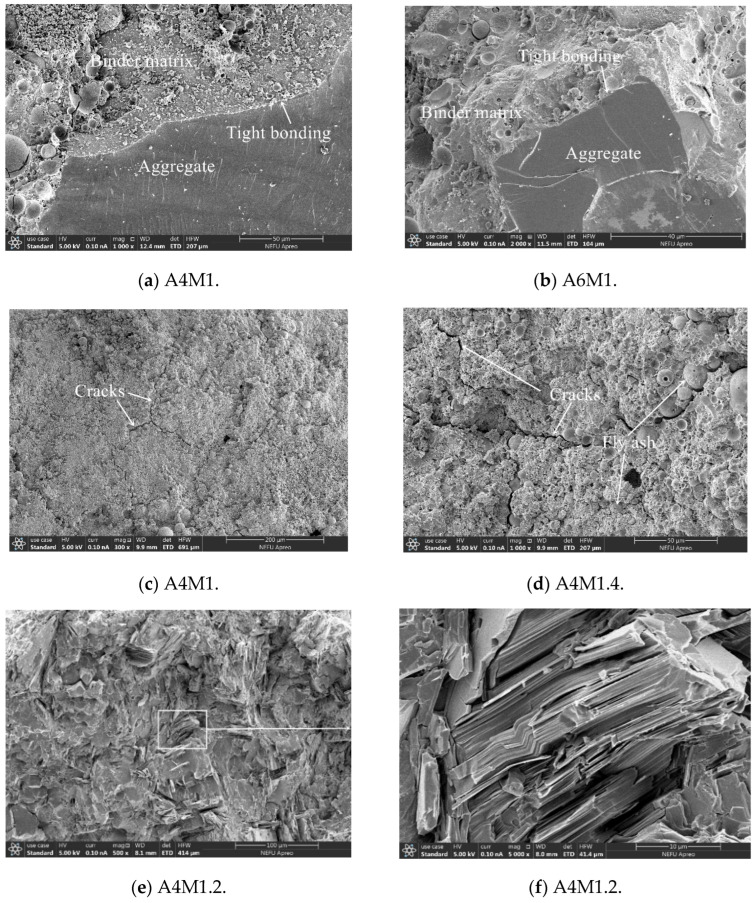
SEM images of AAFSC.

Figure 10 shows the SEM images of AAFSC. In Figure 10a,b, the alkali-activated cementitious matrix structure of A4M1 and A6M1 was dense and had good bonding with the aggregate. The authors have conducted a systematic phase analysis of alkali-activated fly ash slag pastes in preliminary research [27]. By analyzing the phase combination evolution and microstructure of AAFSC [42,43,44,45,46], it was found that the calcium-based hydration product C-A-S-H coexisted with the polymerization product N-A-S-H, making the alkali-activated cementitious matrix denser, with smaller cracks and pore spaces [43], and reducing the porosity in the interface transition zone, thus forming a dense microstructure [45]. Due to the ideal micromechanical properties and denser microstructure of AAFSC compared to paste matrix, the interface transition zone was not the weakest area in AAFSC. A denser interface transition zone was beneficial for the long-term performance of concrete [45,46].

Similar to the cement concrete in Figure 9b, cracks existed in the alkali-activated cementitious matrix in Figure 10c,d, and unreacted fly ash particles of different sizes could also be observed. In Figure 10e,f, locally generated structurally dense layered products could be observed in the A4M1.2 cementitious matrix.

**Figure 11:**
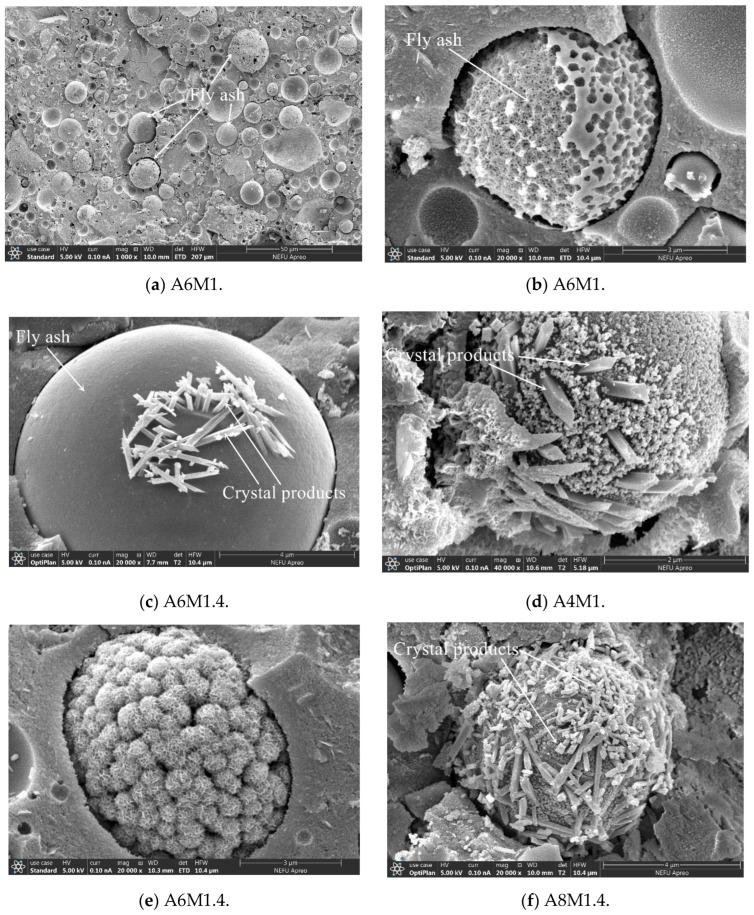
SEM images of fly ash particles in AAFSC.

Figure 11 shows SEM images of fly ash particles with different degrees of reaction in AAFSC. In Figure 11a, there were fly ash particles of different sizes in A6M1, and the reaction degree and product morphology of the fly ash particles were relatively rich. In Figure 11b, most of the fly ash particle in the gel matrix of A6M1 had participated in the reaction, resulting in a hollow product structure. In Figure 11c, the fly ash particle with a small amount of prismatic crystals produced on the surface could be observed when magnified 20,000 times. In Figure 11d, the surface layer of the fly ash particle of A4M1 group completely reacted, producing both the same prismatic crystal as in Figure 11c and the looser gel particles. In Figure 11e, the fly ash particle of the A6M1.4 group concrete completely reacted, producing a large number of spherical hollow structure products piled up together. In Figure 11f, a large number of prismatic crystal structures were formed on the surface of an A8M1.4 fly ash particle. Due to the high content of fly ash in AAFSC and its curing at room temperature, there were still some fly ash particles that had not participated in the reaction or had a low degree of reaction. Based on the previous research, it is believed that the strength of concrete will continue to develop, and the microstructure of alkali-activated cementitious matrix will become denser. After 90 days, most of the reactions will gradually complete.

**Figure 12:**
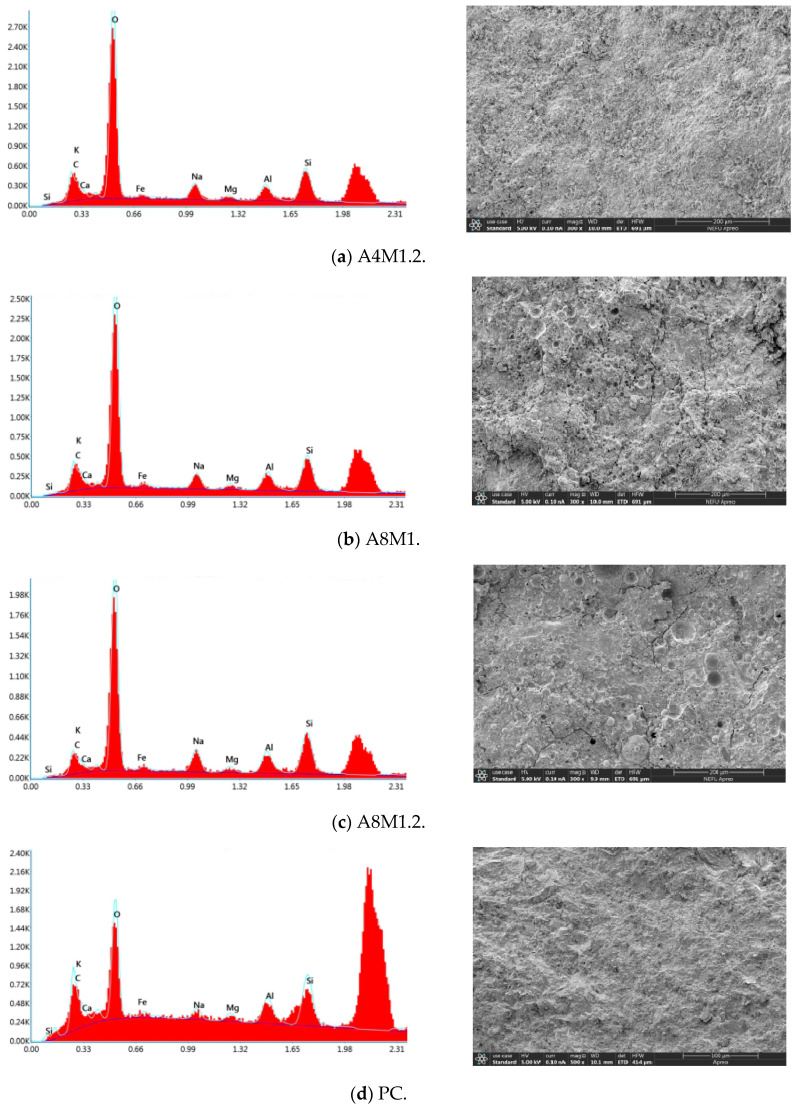
EDX spectra of AAFSC and cement concrete.

Figure 12 shows the EDX spectra of cement concrete and AAFSC. The gel product of AAFSC was mainly composed of Si, Al, O, C, K, and Na, as well as a small amount of Ca, Fe, and Mg elements. The gel product of cement concrete was composed of Si, Al, Fe, O, C, K, and Na, as well as a small amount of Ca and Mg elements. The presence of Na in AAFSC could be explained by the formation of reactants during the condensation process caused by polymerization. These reactants combine with Na ions dissociated from external NaOH, leading to the aggregation of reaction products and enhancing their strength [47]. The Si/Al ratio of AAFSC was approximately between 1.5 and 2, with mainly two types of gel identified: (a) alumino–silicate–hydrate with Na in structure, and (b) calcium–silicate–hydrate with Na in structure [48,49,50].

**Figure 13:**
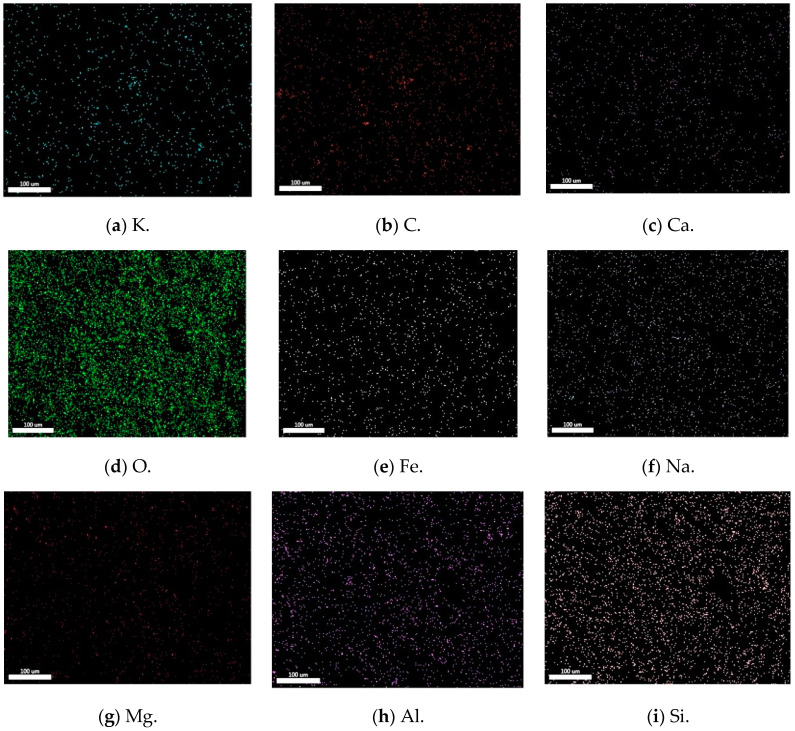
Element distribution map of A6M1.

Figure 13 shows the element distribution map of A6M1, with significant differences in the distribution of different elements. Among them, O and Si elements have the most obvious distribution. Table 8 shows the EDX element content statistics of AAFSC and cement concrete. It can be seen that the elemental composition of AAFSC with different mix ratios was not significantly different. This is because the EDX scanning points had almost no coarse aggregates, while the cementitious components of AAFSC with different mix ratios were the same, both consisting of 80% fly ash and 20% slag, with the difference being the composition of alkali activators.

In contrast, the elemental composition of AAFSC was significantly different from that of cement concrete. The content of O element was much higher, while the content of other elements such as Fe, Mg, and Al was significantly lower than that of cement concrete. The Ca/Si value of AAFSC was smaller than that of cement concrete. Generally, the lower the Ca/Si value, the denser the concrete structure. This may be one of the reasons why the AAFSC had better water absorption and impermeability performance than cement concrete in this study.

## 6. Conclusions

This article systematically studies the mechanical properties, durability, and microstructure of AAFSC with fly ash content accounting for 80% of the total precursor under room-temperature curing conditions. The effects of alkali equivalent and activator modulus on the compressive strength, impermeability, water absorption, and microstructure of AAFSC were studied and compared with cement concrete. The following main conclusions were drawn:

1. The 7-day compressive strength range of AAFSC was from 18 MPa to 25.2 MPa, all lower than that of cement concrete. The 28-day compressive strength ranged from 30.4 MPa to 38 MPa, all higher than that of the cement concrete. The 28-day compressive strength of A8M1 reached 38 MPa, which was 131.94% of the value of cement concrete.
2. In this study, AAFSC exhibited excellent impermeability protection performance. The air permeability coefficient increased first and then decreased with the increase of alkali equivalent, and decreased with the decreasing alkali activator modulus. Among them, the AAFSC with activator modulus of 1.0 had better impermeability than the cement concrete.
3. The water absorption curves of AAFSC were entirely below that of cement concrete, indicating a lower water absorption rate. The water absorption rate of AAFSC decreased with the increasing alkali equivalent, and the value of A8M1 was 2.13%, which was only 60.86% of that of cement concrete. The water absorption model established could predict the water absorption capacity of AAFSC at different water absorption stages in this study.
4. Through SEM and EDX analysis, it was found that the cementitious matrix of AAFSC was well bonded to the aggregate, and fly ash particles with different degrees of reaction could be seen, indicating that the reaction of fly ash was still ongoing. The Ca/Si value of AAFSC was lower, which may be one of the reasons why the AAFSC had better water absorption and impermeability performance than cement concrete in this study.

Outlook: Previous studies have confirmed that short cut fibers, such as glass fiber and polypropylene fiber, can significantly improve the mechanical properties and durability of ordinary cement concrete. However, the improvement effect of these fibers on the performance of AAFSC needs further research.

## Figures and Tables

**Table 1:** Chemical components of fly ash [27].

Chemical Components	Content (wt%)
SiO_2_	58.23
Al_2_O_3_	19.21
Fe_2_O_3_	8.2
CaO	7.12
K_2_O	2.29
MgO	1.46
Na_2_O	1.32
TiO_2_	0.78
SO_3_	0.54
P_2_O_5_	0.25
Other Components	0.6

**Table 2:** Chemical components of slag [27].

Chemical Components	Content (wt%)
CaO	40.63
SiO_2_	29.51
Al_2_O_3_	15.32
MgO	7.82
SO_3_	2.74
TiO_2_	1.40
Fe_2_O_3_	0.85
MnO	0.53
K_2_O	0.46
Na_2_O	0.41
Other Components	0.33

**Table 3:** Mix proportions of AAFSC.

MIX ID	Fly Ash (kg/m^3^)	Slag (kg/m^3^)	Sand (kg/m^3^)	Stone (kg/m^3^)	Sodium Silicate (kg/m^3^)	Sodium Hydroxide (kg/m^3^)	Water (kg/m^3^)	Water Reducer (kg/m^3^)
A4M1	344	86	655	1165	54.5	12.3	120.4	3.01
A6M1	344	86	655	1165	81.7	18.4	105.3	3.01
A8M1	344	86	655	1165	109	24.6	90.2	3.01
A4M1.2	344	86	655	1165	65.4	10.3	114.3	3.01
A6M1.2	344	86	655	1165	98.1	15.5	96.2	3.01
A8M1.2	344	86	655	1165	130.8	20.6	78.2	3.01
A4M1.4	344	86	655	1165	76.3	8.3	108.3	3.01
A6M1.4	344	86	655	1165	114.4	12.5	87.2	3.01
A8M1.4	344	86	655	1165	152.6	16.7	66.1	3.01

**Table 4:** Mix proportion of cement concrete.

MIX ID	Cement (kg/m^3^)	Fly Ash (kg/m^3^)	Silica Fume (kg/m^3^)	Slag (kg/m^3^)	Sand (kg/m^3^)	Stone (kg/m^3^)	Water (kg/m^3^)	Water Reducer (kg/m^3^)
PC	280	86	22	43	655	1165	129	6.45

**Table 5:** Compressive strength of concrete.

**MIX ID**	**7-Day Compressive Strength (MPa)**	**Standard Deviation**	**28-Day Compressive Strength (MPa)**	**Standard Deviation**
PC	28.6	0.78	28.8	2.12
A4M1	22.4	2.16	34.9	0.72
A6M1	25.1	0.52	37.5	1.06
A8M1	25.2	0.8	38	0.72
A4M1.2	18	1.64	30.4	1.04
A6M1.2	24.7	0.4	35.3	2.13
A8M1.2	24.8	1.63	35.4	1.51
A4M1.4	18.9	0.24	30.2	0.55
A6M1.4	24.7	1.19	36.7	1.08
A8M1.4	19.2	1.13	31	1.11

**Table 6:** Air permeability coefficient and protection quality level of concrete.

MIX ID	Air Permeability Coefficient	Protection Quality Level
PC	1.691 × 10^−16^	Good
A4M1	0.957 × 10^−16^	Very good
A6M1	1.471 × 10^−16^	Good
A8M1	1.084 × 10^−16^	Very good
A4M1.2	2.401 × 10^−16^	Good
A6M1.2	3.204 × 10^−16^	Good
A8M1.2	2.181 × 10^−16^	Good
A4M1.4	2.264 × 10^−16^	Good
A6M1.4	3.246 × 10^−16^	Good
A8M1.4	2.229 × 10^−16^	Good

**Table 7:** Fitting parameters of the long-term water absorption model for concrete.

MIX ID	φ	ω	*R* ^2^
A4M1	2.5388	2.6516	0.9955
A6M1	2.2997	2.0327	0.9953
A8M1	2.0696	1.1340	0.9728
A4M1.2	3.2751	3.2962	0.9973
A6M1.2	2.6705	2.8864	0.9979
A8M1.2	2.1599	1.4767	0.9889
A4M1.4	3.0700	3.2757	0.9967
A6M1.4	2.5386	3.1851	0.9964
A8M1.4	2.3010	1.9152	0.9941
PC	3.4595	1.8585	0.9917

**Table 8:** EDX element composition of AAFSC and cement concrete.

Mix ID	Element (wt%)									
	O	Si	Al	Fe	Na	Mg	K	C	Ca	Ca/Si
A4M1	47	14	8	5	8	4	4	5	4	0.286
A6M1	44	15	10	5	8	4	4	5	4	0.267
A8M1	45	15	9	5	8	4	4	5	4	0.267
A4M1.2	47	15	8	5	8	4	4	6	4	0.267
A6M1.2	42	17	10	6	8	5	4	5	4	0.235
A8M1.2	45	17	9	5	9	4	3	4	3	0.176
A4M1.4	48	16	8	5	8	4	3	5	4	0.250
A6M1.4	52	15	8	4	8	3	3	4	3	0.200
A8M1.4	48	16	8	5	9	4	3	4	4	0.250
PC	25	17	13	9	9	8	5	7	5	0.294

## Data Availability

The original contributions presented in this study are included in the article. Further inquiries can be directed to the corresponding author.

## References

[1] Joseph B., Mathew G. (2012). Influence of aggregate content on the behavior of fly ash based geopolymer concrete. Entia Iran..

[2] Rafeet A., Vinai R., Soutsos M., Sha W. (2019). Effects of slag substitution on physical and mechanical properties of fly ash-based alkali activated binders (AABs). Cem. Concr. Res..

[3] Nath P., Sarker P.K. (2014). Effect of GGBFS on setting, workability and early strength properties of fly ash geopolymer concrete cured in ambient condition. Constr. Build. Mater..

[4] Rafeet A., Vinai R., Soutsos M., Sha W. (2017). Guidelines for mix proportioning of fly ash/GGBS based alkali activated concretes. Constr. Build. Mater..

[5] Deb P.S., Nath P., Sarker P.K. (2014). The effects of ground granulated blast-furnace slag blending with fly ash and activator content on the workability and strength properties of geopolymer concrete cured at ambient temperature. Mater. Des..

[6] Athira V.S., Bahurudeen A., Saljas M., Jayachandran K. (2021). Influence of different curing methods on mechanical and durability properties of alkali activated binders. Constr. Build. Mater..

[7] Kim T., Kang C. (2021). Investigation of the Effect of Mixing Time on the Mechanical Properties of Alkali-Activated Cement Mixed with Fly Ash and Slag. Materials.

[8] Alrefaei Y., Wang Y.S., Dai J.G. (2021). Effect of mixing method on the performance of alkali-activated fly ash/slag pastes along with polycarboxylate admixture. Cem. Concr. Compos..

[9] Jang J.G., Lee N.K., Lee H.K. (2014). Fresh and hardened properties of alkali-activated fly ash/slag pastes with superplasticizers. Constr. Build. Mater..

[10] de Hita M.J., Criado M. (2022). Influence of the Fly Ash Content on the Fresh and Hardened Properties of Alkali-Activated Slag Pastes with Admixtures. Materials.

[11] Nedeljković M., Ghiassi B., van der Laan S., Li Z., Ye G. (2018). Effect of curing conditions on the pore solution and carbonation resistance of alkali-activated fly ash and slag pastes. Cem. Concr. Res..

[12] Yang T., Zhu H., Zhang Z., Gao X., Zhang C., Wu Q. (2018). Effect of fly ash microsphere on the rheology and microstructure of alkali-activated fly ash/slag pastes. Cem. Concr. Res..

[13] Wang Y., Cao Y., Ma Y., Xiao S., Hu J., Wang H. (2021). Fresh and hardened properties of alkali-activated fly ash/slag binders: Effect of fly ash source, surface area, and additives. J. Sustain. Cem.-Based Mater..

[14] El-Hassan H., Shehab E., Al-Sallamin A. (2021). Effect of curing regime on the performance and microstructure characteristics of alkali-activated slag-fly ash blended concrete. J. Sustain. Cem.-Based Mater..

[15] Rodrigue A., Duchesne J., Fournier B., Bissonnette B. (2018). Influence of added water and fly ash content on the characteristics, properties and early-age cracking sensitivity of alkali-activated slag/fly ash concrete cured at ambient temperature. Constr. Build. Mater..

[16] Aiken T.A., Kwasny J., Sha W., Tong K.T. (2021). Mechanical and durability properties of alkali-activated fly ash concrete with increasing slag content. Constr. Build. Mater..

[17] Bondar D., Basheer M., Nanukuttan S. (2019). Suitability of alkali activated slag/fly ash (AA-GGBS/FA) concretes for chloride environments: Characterisation based on mix design and compliance testing. Constr. Build. Mater..

[18] Lee N.K., Lee H.K. (2013). Setting and mechanical properties of alkali-activated fly ash/slag concrete manufactured at room temperature. Constr. Build. Mater..

[19] Aliques-Granero J., Tognonvi M.T., Tagnit-Hamou A. (2019). Durability study of AAMs: Sulfate attack resistance. Constr. Build. Mater..

[20] Mukherjee S., Shi X., Deng Y., Udpa L. (2020). A Hybrid Microwave NDE System for Rapid Inspection of GFRP Composites. Mater. Eval..

[21] Shi X., Olvera A., Hamilton C., Gao E., Li J., Utke L., Petruska A., Yu Z., Udpa L., Deng Y. (2021). AI-Enabled Robotic NDE for Structural Damage Assessment and Repair. Mater. Eval..

[22] Shi X., Rathod V.T., Mukherjee S., Udpa L., Deng Y. (2020). Multi-modality strain estimation using a rapid near-field microwave imaging system for dielectric materials. Measurement.

[23] Wang J., Xie J., Wang C., Zhao J., Liu F., Fang C. (2020). Study on the optimum initial curing condition for fly ash and GGBS based geopolymer recycled aggregate concrete. Constr. Build. Mater..

[24] Xie J., Wang J., Zhang B., Fang C., Li L. (2019). Physicochemical properties of alkali activated GGBS and fly ash geopolymeric recycled concrete. Constr. Build. Mater..

[25] Rodrigue A., Duchesne J., Fournier B., Champagne M., Bissonnette B. (2020). Alkali-silica reaction in alkali-activated combined slag and fly ash concretes: The tempering effect of fly ash on expansion and cracking. Constr. Build. Mater..

[26] Keulen A., Yu Q.L., Zhang S., Grünewald S. (2018). Effect of admixture on the pore structure refinement and enhanced performance of alkali-activated fly ash-slag concrete. Constr. Build. Mater..

[27] Yuan Z., Jia Y., Sun J., Zhang X., Hu Y., Han X. (2024). Study on the Properties of High Fly Ash Content Alkali-Activated Fly Ash Slag Pastes and Fiber-Reinforced Mortar Under Normal Temperature Curing. Materials.

[28] Yuan Z., Jia Y. (2020). Mechanical properties and microstructure of glass fiber and polypropylene fiber reinforced concrete: An experimental study. Constr. Build. Mater..

[29] Provis J.L., Deventer J. (2014). Alkali Activated Materials.

[30] Escalante García J.I., Campos-Venegas K., Gorokhovsky A., Fernández A. (2006). Cementitious composites of pulverised fuel ash and blast furnace slag activated by sodium silicate: Effect of Na_2_O concentration and modulus. Adv. Appl. Ceram..

[31] (2019). Standard for Test Method of Mechanical Properties on Ordinary Concrete.

[32] (2013). Standard Test Method for Density, Absorption, and Voids in Hardened Concrete, Annual Book of ASTM Standard.

[33] (2010). Standard Guide for Examination of Hardened Concrete Using Scanning Electron Microscopy, Annual Book of ASTM Standard.

[34] Hannawi K., Bian H., Prince-Agbodjan W., Raghavan B. (2016). Effect of different types of fibers on the microstructure and the mechanical behavior of Ultra-High Performance Fiber-Reinforced Concretes. Compos. Part B.

[35] Yu Z., Ni C., Tang M., Shen X. (2018). Relationship between water permeability and pore structure of Portland cement paste blended with fly ash. Constr. Build. Mater..

[36] Behfarnia K., Behravan A. (2014). Application of high performance polypropylene fibers in concrete lining of water tunnels. Mater. Des..

[37] Powers T.C. (1945). A working hypothesis for further studies of frost resistance of concrete. J. Am. Concr. Inst..

[38] Izaguirre A., Lanas J., Alvarez J. (2010). Effect of a polypropylene fibre on the behaviour of aerial lime-based mortars. Constr. Build. Mater..

[39] Fallah S., Nematzadeh M. (2017). Mechanical properties and durability of high-strength concrete containing macro-polymeric and polypropylene fibers with nano-silica and silica fume. Constr. Build. Mater..

[40] Yuan Z., Jia Y. (2022). Experimental Study on the Mechanical Properties, Water Absorption, and Fiber Degradation of Naturally Aged Glass Fiber and Polypropylene Fiber-Reinforced Concrete. Materials.

[41] Afroughsabet V., Ozbakkaloglu T. (2015). Mechanical and durability properties of high-strength concrete containing steel and polypropylene fibers. Constr. Build. Mater..

[42] Radlinska A.H. (2016). Fly ash-slag interaction during alkaline activation: Influence of activators on phase assemblage and microstructure formation. Constr. Build. Mater..

[43] Mehta A., Siddique R., Ozbakkaloglu T., Shaikh F.U.A., Belarbi R. (2020). Fly ash and ground granulated blast furnace slag-based alkali-activated concrete: Mechanical, transport and microstructural properties. Constr. Build. Mater..

[44] Celikten S., Saridemir M., Deneme I.O. (2019). Mechanical and microstructural properties of alkali-activated slag and slag + fly ash mortars exposed to high temperature. Constr. Build. Mater..

[45] Fang G., Zhang M. (2019). The evolution of interfacial transition zone in alkali-activated fly ash-slag concrete. Cem. Concr. Res..

[46] Fang G., Wang Q., Zhang M. (2021). Micromechanical analysis of interfacial transition zone in alkali-activated fly ash-slag concrete. Cem. Concr. Compos..

[47] Ryu G.S., Lee Y.B., Koh K.T., Chung Y.S. (2013). The mechanical properties of fly ash-based geopolymer concrete with alkaline activators. Constr. Build. Mater..

[48] Kumar S., Kumar R., Mehrotra S.P. (2010). Influence of granulated blast furnace slag on the reaction, structure and properties of fly ash based geopolymer. J. Mater. Sci..

[49] Singh B., Rahman M.R., Paswan R., Bhattacharyya S.K. (2016). Effect of activator concentration on the strength, ITZ and drying shrinkage of fly ash/slag geopolymer concrete. Constr. Build. Mater..

[50] Bernal S.A., Provis J.L., Walkley B., San Nicolas R., Gehman J.D., Brice D.G., Kilcullen A.R., Duxson P., van Deventer J.S. (2013). Gel nanostructure in alkali-activated binders based on slag and fly ash, and effects of accelerated carbonation. Cem. Concr. Res..

